# Supplementary figures and images for: Functional Specialization of S-Adenosylmethionine Synthases Links Phosphatidylcholine to Mitochondrial Function and Stress Survival
Source: bioRxiv. 2025 Feb 22:2025.02.20.639242. Preprint. [Version 2] doi: 10.1101/2025.02.20.639242 (PMC11870525; doi:10.1101/2025.02.20.639242)

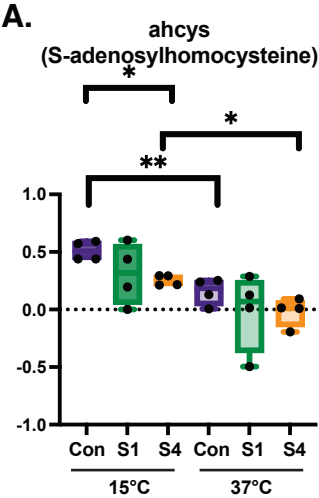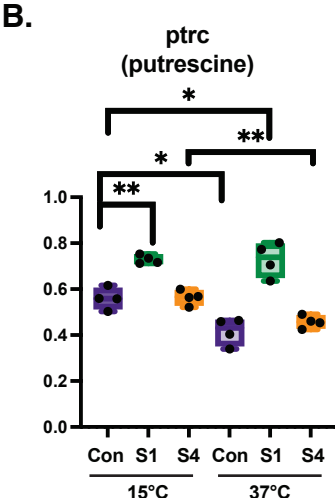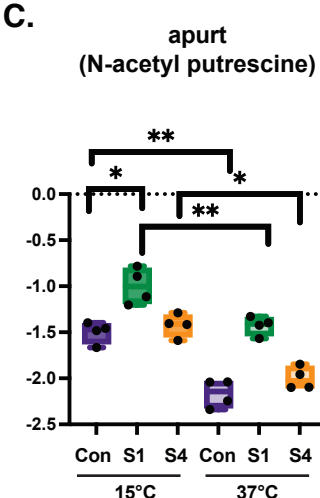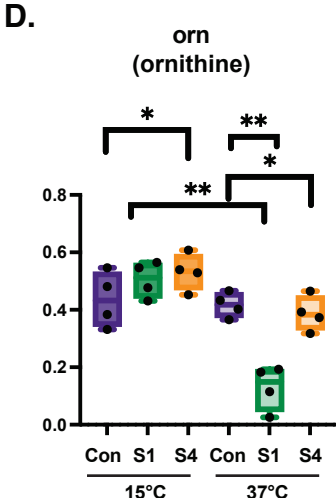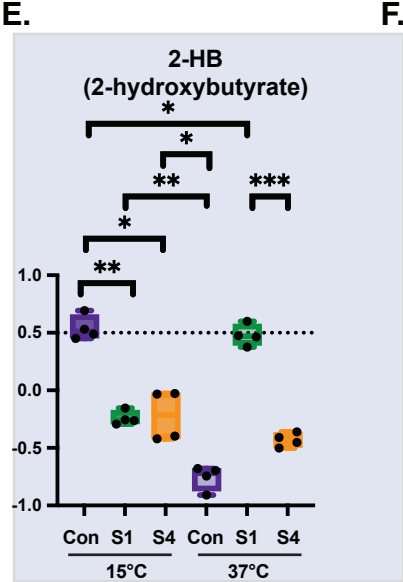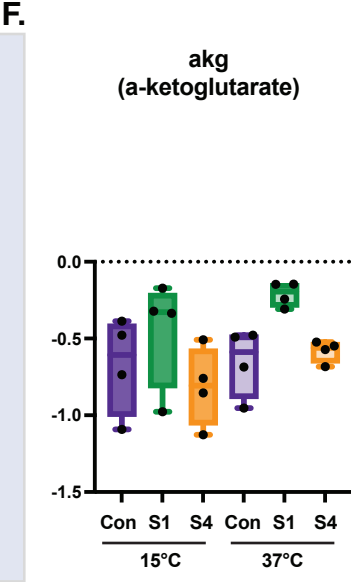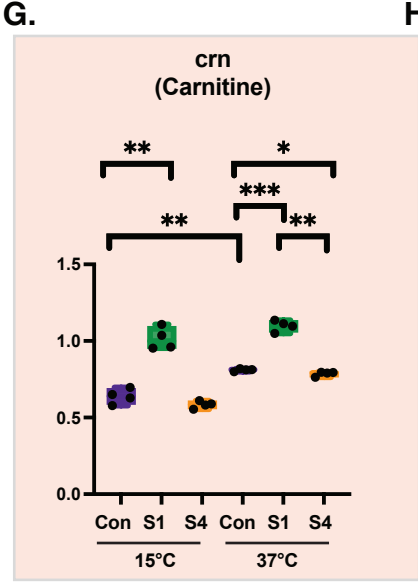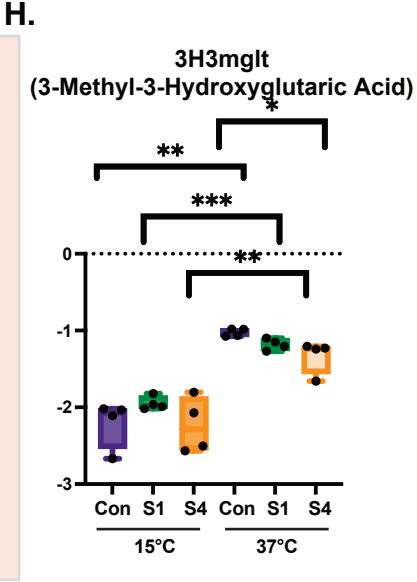

Supplement: Supplement 5 — Figure S2: Comparison of individual metabolite levels from targeted metabolomics. Box and whisker plots showing individual metabolites from targeted metabolomics comparing heat shocked sams-1 and sams-4(RNAi) animals (A-H). Colored boxes show location of selected metabolites on Figure S2. Significance was determined by two-way repeated measures ANOVA. ns: q-value ≥ 0.05 *: q-value < 0.05, **: q-value < 0.01, ***: q-value < .001, ****: q-value > 0.0001. Color blocks map to areas on metabolic map (Figure S2, Figure 2 and Figure 3). [file media-5.pdf]

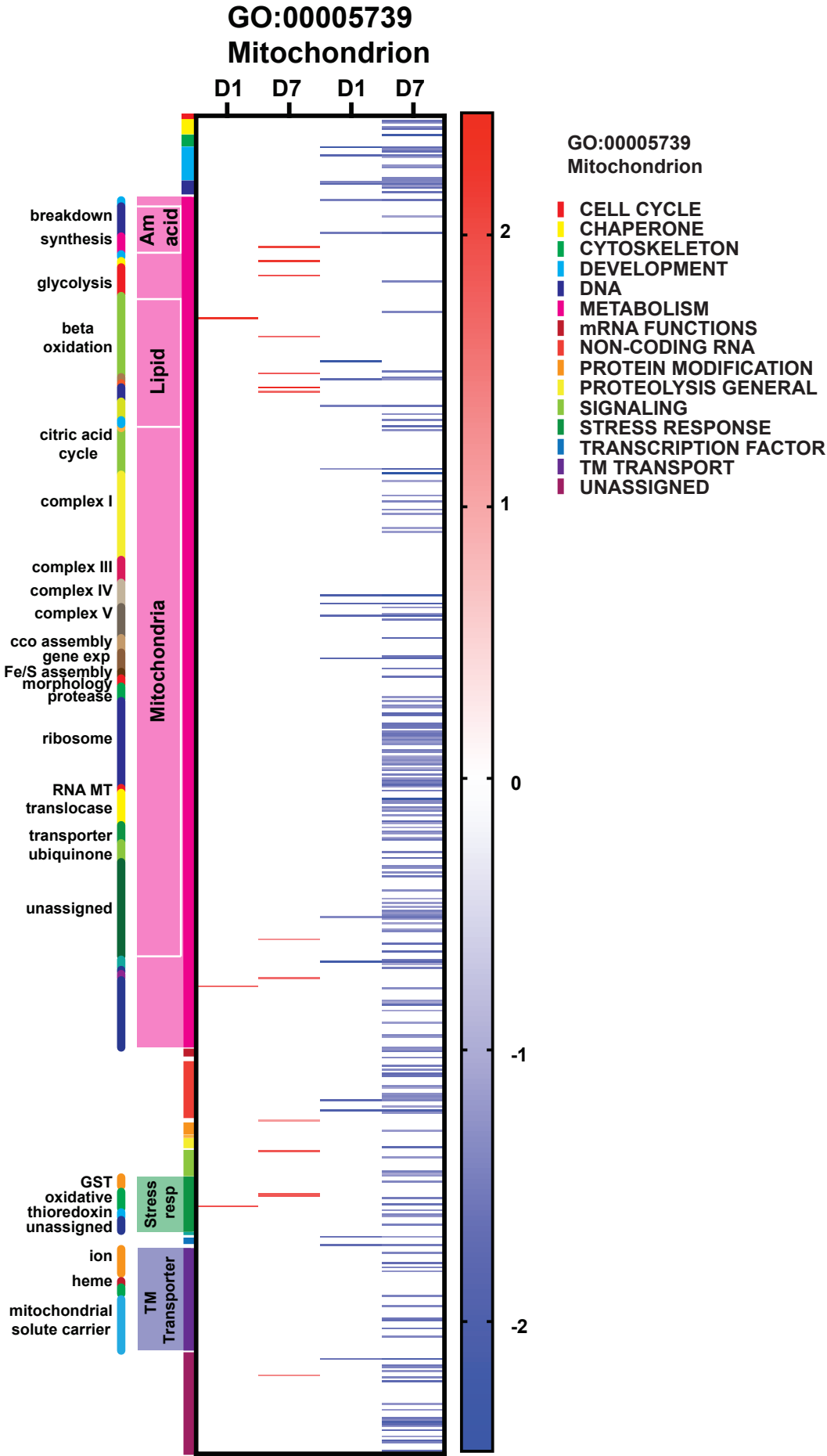

Supplement: Supplement 6 — Figure S3: Distribution of sams-1(lof) upregulated genes during aging in GO. Heat map of GO: 00005739 (Mitochondrion) category. [file media-6.pdf]

A.

*hlh-30*

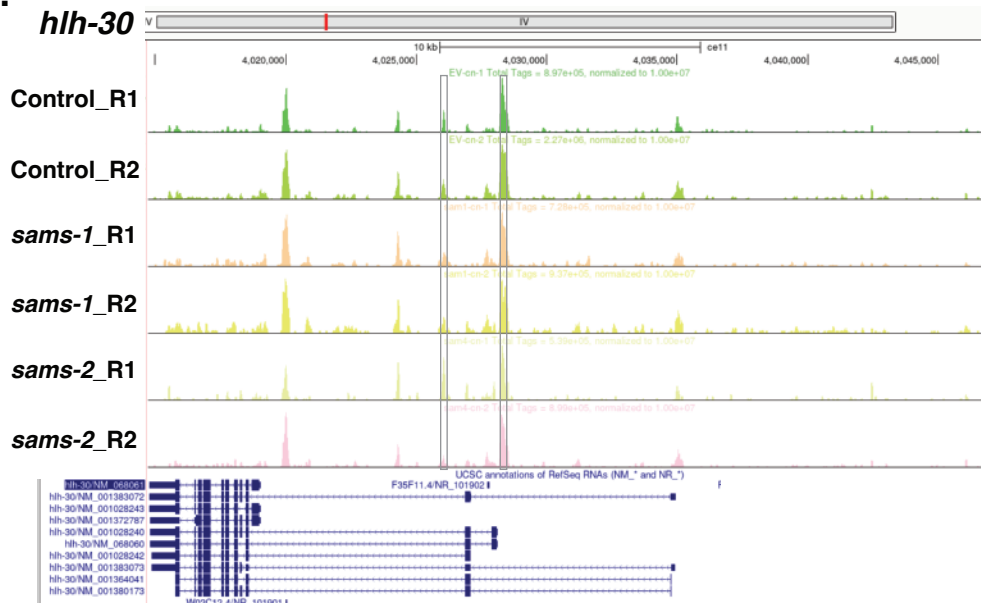

B.

*pha-4*

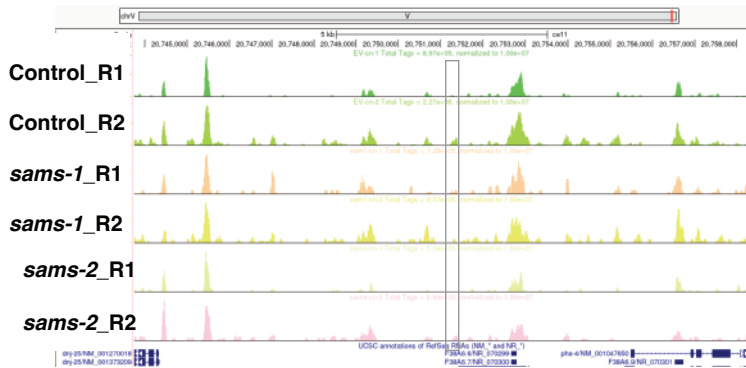

C.

*sqst-1*

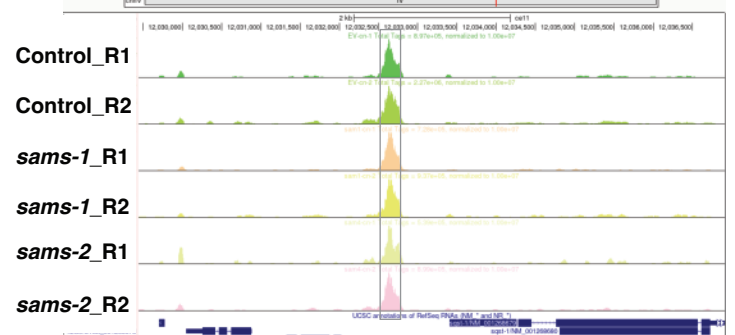

D.

*lgg-1*

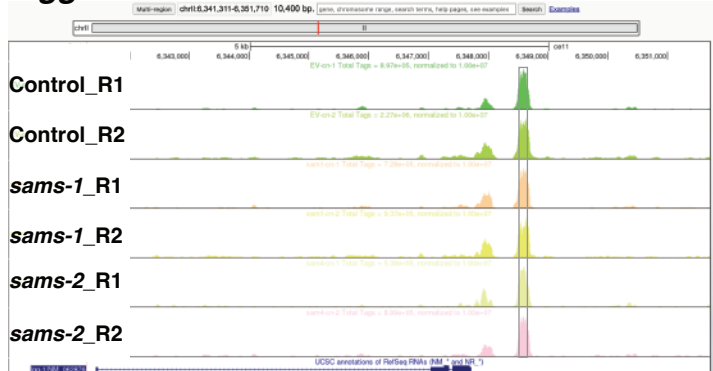

E.

*lgg-2*

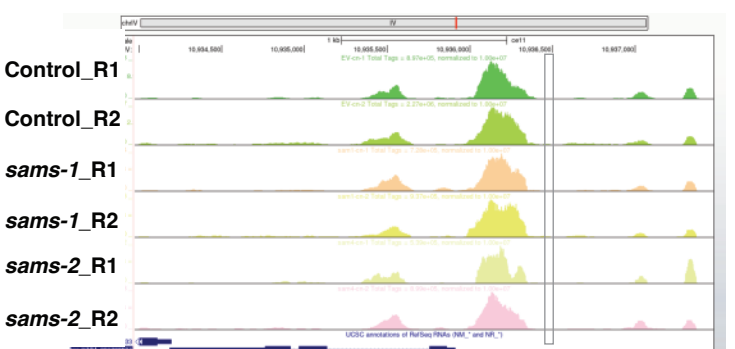

F.

*atg-4.1*

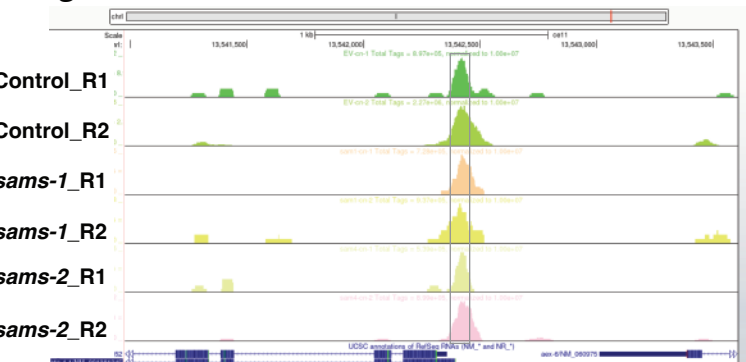

G.

*sodh-1*

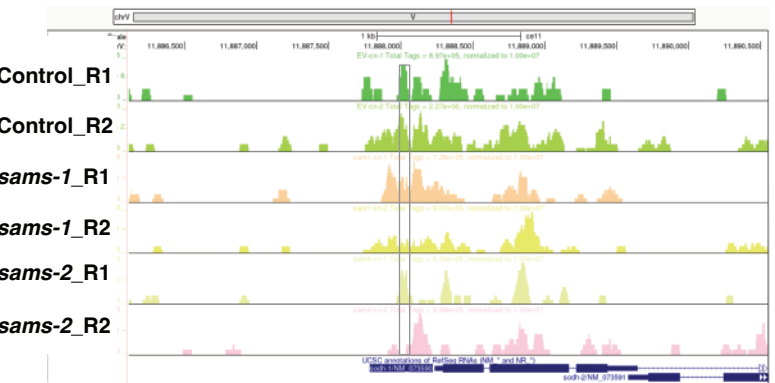

Supplement: Supplement 7 — Figure S4: Browser tracks showing H3K4me3 peaks from Godbole, et al. eLife (2023) of autophagy-related genes after sams-1 and sams-4(RNAi) for hlh-30 (A), pha-4 (B), sqst-1(C), lgg-1 (D), lgg-2 (E), atg-4.1 (F), and sodh-1(G). Locations for primer sets used in Lim et al 2023 are boxed. [file media-7.pdf]

A.

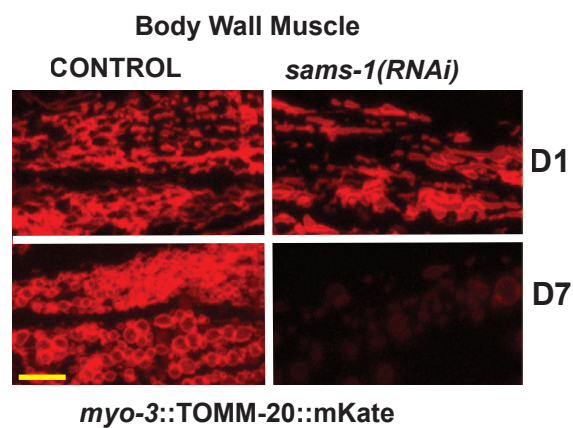

B.

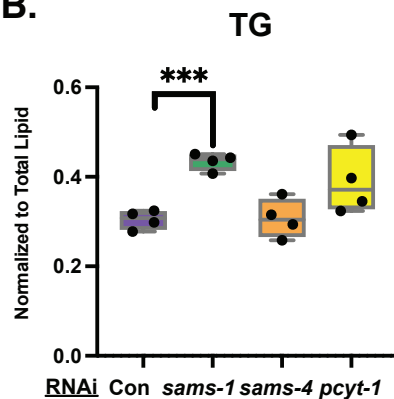

C.

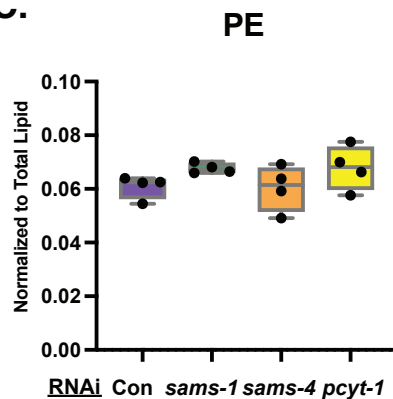

D.

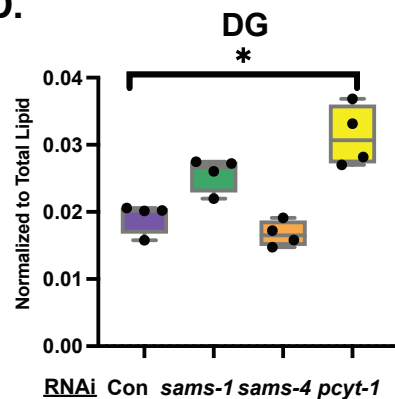

E.

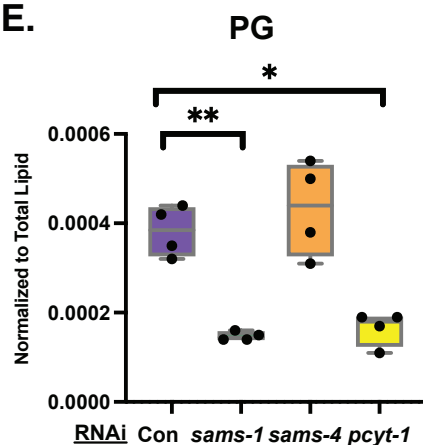

F.

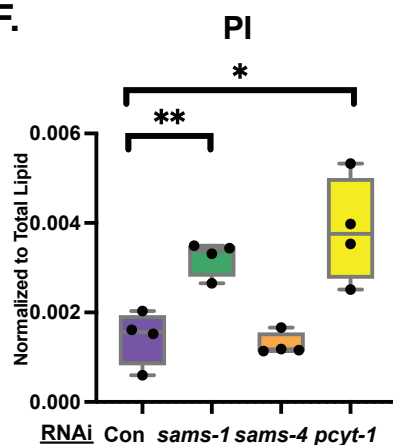

G.

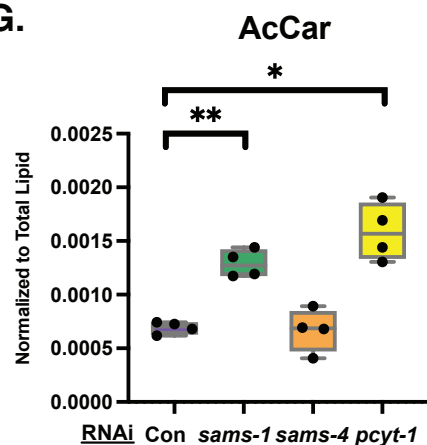

H.

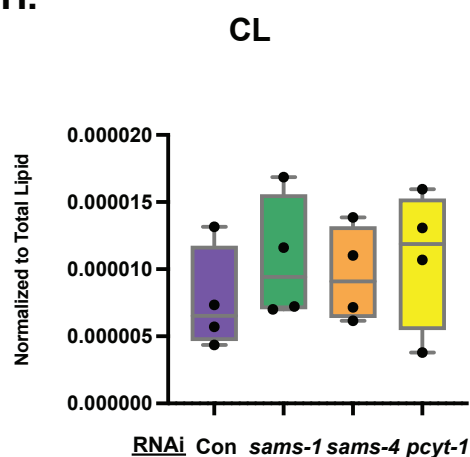

I.

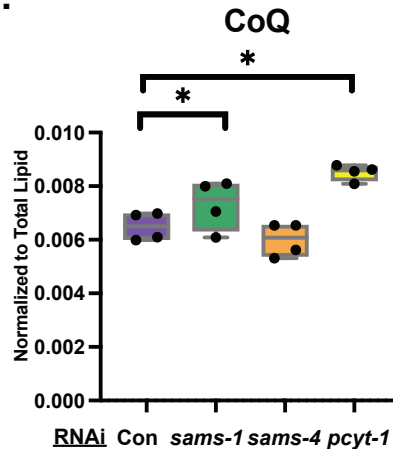

J.

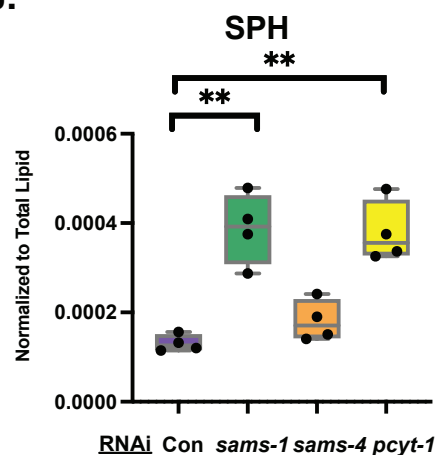

Supplement: Supplement 8 — Figure S5: Changes in mitochondrial morphology and lipid levels occur after loss of sams-1. A. Confocal projection shows mitochondrial fission progression to loss from D1 to D7 in body wall muscle expressing TOMM-20::mKATE animals after sams-1(RNAi). LCMS analysis comparing lipid class levels after sams-1, sams-4 or pcyt-1(RNAi) for TG (triglycerides) (B), PE (phosphatidylethanolamines) (C), diglycerides (DG, D), phosphatidylglycerols (PG, E), Phosphatidylinstitols (PI, F), Acylcarnatines (AcCa, G), cardiolipins (CL, H), Ubiquinone isoforms (CoQ, I) and sphingolipids (SPH, J). [file media-8.pdf]

A.

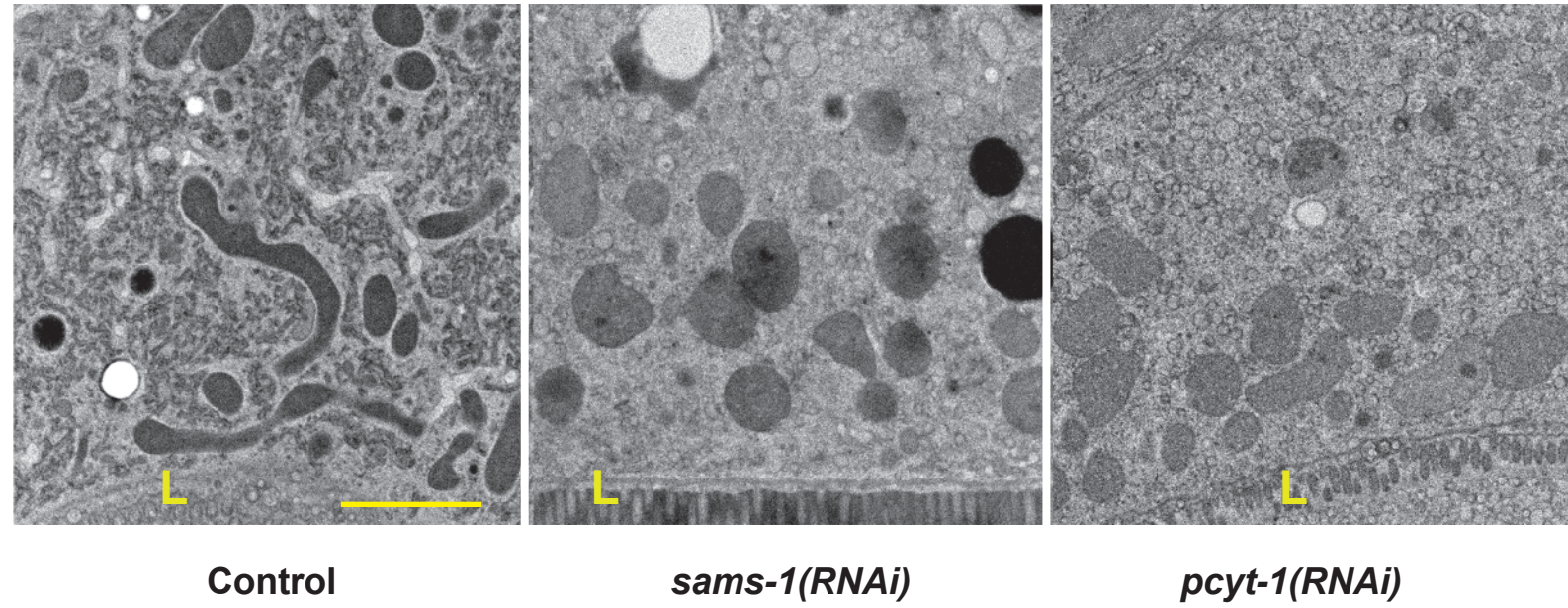

B.

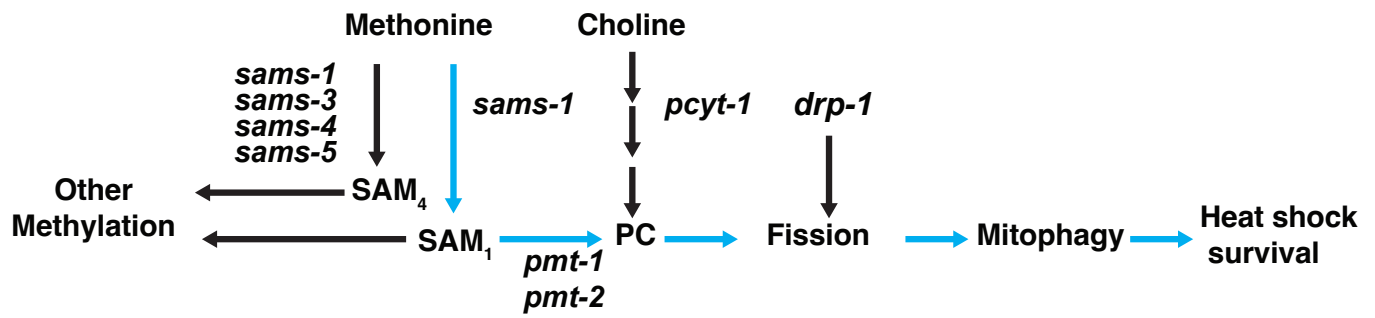

Supplement: Supplement 9 — Figure S6. Changes in mitochondrial morphology and cellular localization occur after loss of sams-1. A. TEM images of Control, sams-1 or pcyt-1 RNAi animals comparing mitochondrial morphology, localization and localization within intestinal cells. Scale bar is 500 nm. B. Model. [file media-9.pdf]
