# Supplementary material for: Functional Specialization of S-Adenosylmethionine Synthases Links Phosphatidylcholine to Mitochondrial Function and Stress Survival
Source: bioRxiv. 2025 Feb 22:2025.02.20.639242. Preprint. [Version 2] doi: 10.1101/2025.02.20.639242 (PMC11870525; doi:10.1101/2025.02.20.639242)

Figure S1
Munden, et al.

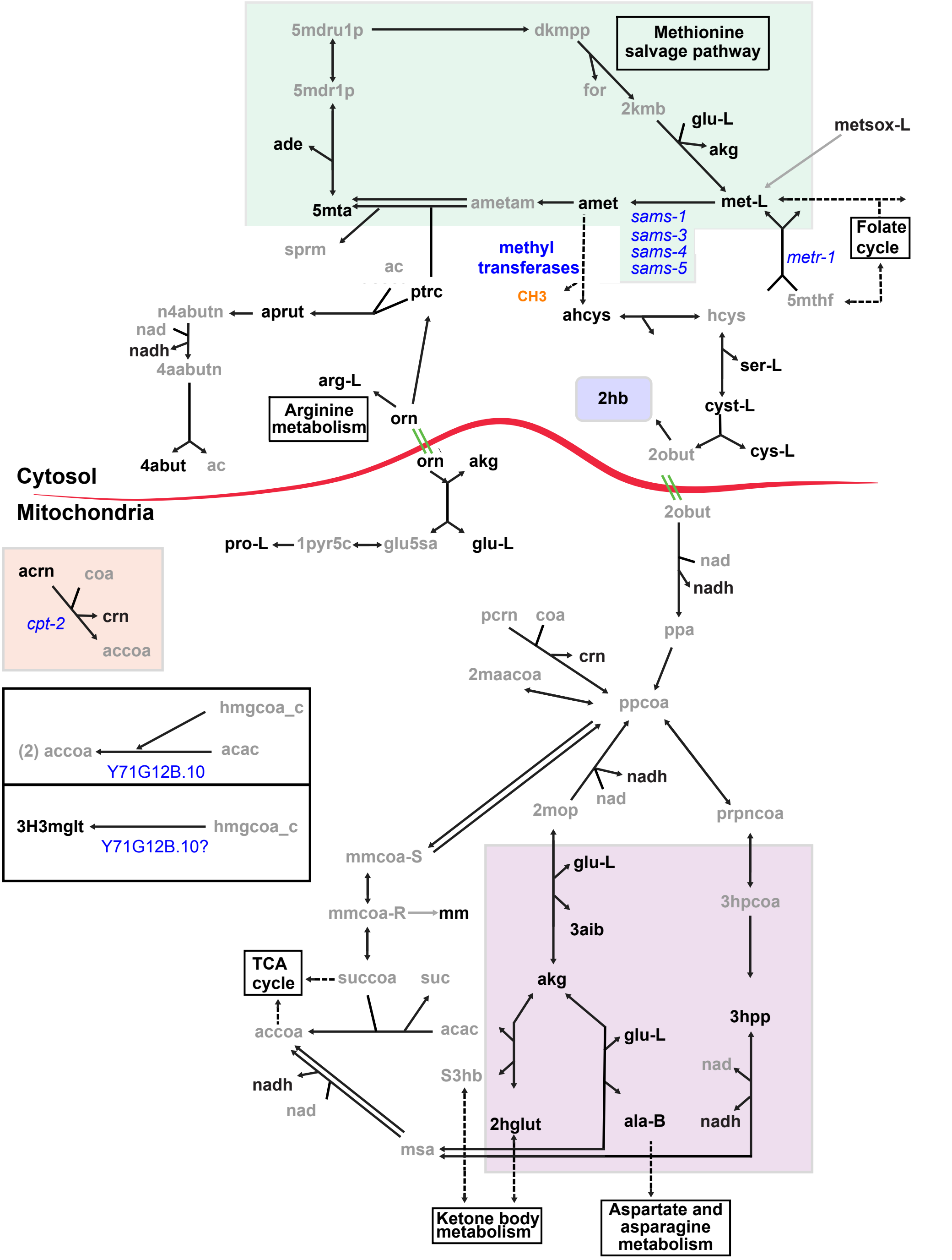

Supplement: Supplement 4 — Figure S1: Schematic of metabolic pathways represented in data from targeted metabolomics comparing SAM synthase knockdown in basal and heat shocked C. elegans, based on WormPaths diagrams. Pathways were adapted from WormPaths (16). Colored boxes correspond to metabolites shown in individual graphs. Bolded metabolites are represented in targeted metabolomics. [file media-4.pdf]
